# What’s under the Christmas Tree? A Soil Sulfur Amendment Lowers Soil pH and Alters Fir Tree Rhizosphere Bacterial and Eukaryotic Communities, Their Interactions, and Functional Traits

**DOI:** 10.1128/spectrum.00166-21

**Published:** 2021-07-07

**Authors:** Blaire Steven, Jacquelyn C. LaReau, Stephen J. Taerum, Nubia Zuverza-Mena, Richard S. Cowles

**Affiliations:** a Department of Environmental Sciences, Connecticut Agricultural Experiment Station, New Haven, Connecticut, USA; b Department of Plant Pathology and Ecology, Connecticut Agricultural Experiment Station, New Haven, Connecticut, USA; c Department of Analytical Chemistry, Connecticut Agricultural Experiment Station, New Haven, Connecticut, USA; d Valley Laboratory, Connecticut Agricultural Experiment Station, Windsor, Connecticut, USA; University of Minnesota

**Keywords:** 16S rRNA, 18S rRNA, Christmas tree, fir tree, soil acidification, metagenome, pH, rhizosphere-inhabiting microbes

## Abstract

In this study, we describe the legacy effects of a soil sulfur amendment experiment performed 6 years prior and the resulting alterations to the rhizosphere communities of fir trees on a Christmas tree plantation. The pH of bulk soil was ∼1.4 pH units lower than that of untreated soils and was associated with reduced Ca, Mg, and organic matter contents. Similarly, root chemistry differed due to the treatment, with roots in sulfur-amended soils showing significantly higher Al, Mn, and Zn contents and reduced levels of B and Ca. 16S rRNA and 18S rRNA gene sequencing was pursued to characterize the bacterial/archaeal and eukaryotic communities in the rhizosphere soils. The treatment induced dramatic and significant changes in the microbial populations, with thousands of 16S rRNA gene sequence variants and hundreds of 18S rRNA gene variants being significantly different in relative abundances between the treatments. Additionally, co-occurrence networks showed that bacterial and eukaryotic interactions, network topology, and hub taxa were significantly different when constructed from the control and treated soil 16S and 18S rRNA gene amplicon libraries. Metagenome sequencing identified several genes related to transport proteins that differentiated the functional potentials of the communities between treatments, pointing to physiological adaptations in the microbial communities for living at altered pH. These data show that a legacy of soil acidification increased the heterogeneity of the soil communities as well as decreasing taxon connections, pointing to a state of ecosystem instability that has potentially persisted for 6 years.

**IMPORTANCE** We used sulfur incorporation to investigate the legacy effects of lowered soil pH on the bacterial and eukaryotic populations in the rhizosphere of Christmas trees. Acidification of the soils drove alterations of fir tree root chemistry and large shifts in the taxonomic and functional compositions of the communities. These data demonstrate that soil pH influences are manifest across all organisms inhabiting the soil, from the host plant to the microorganisms inhabiting the rhizosphere soils. Thus, this study highlights the long-lasting influence of altering soil pH on soil and plant health as well as the status of the microbiome.

## INTRODUCTION

Each year, approximately 33 million to 36 million Christmas trees are produced in North America, with an additional 50 million to 60 million grown in Europe (1). A problem for Christmas tree producers is that fir trees are susceptible to root rot diseases caused by *Phytophthora* species (2, 3). One potential method to control root rot organisms is to alter the soil pH. Reducing the pH has been shown to affect *Phytophthora* by inhibiting the germination and survival of various life stages of the pathogen (4). Fir species of eastern North America are tolerant to low-pH soils, so a program of soil acidification via sulfur amendment was undertaken to investigate the interaction between soil pH and the development of root rot in a Christmas tree plantation. Elemental sulfur was chosen to effect soil acidification, as it is the most efficient material available for this purpose (5). Soil acidification occurs when soil microbes oxidize the sulfur to sulfuric acid (6, 7). Soil pH has been identified as one of the primary soil characteristics that influence the diversity and composition of the indigenous soil microbial communities (8–11).

As plant roots absorb and exchange ions with the soil, the pH at the root surface can often be 1 to 2 pH units different than the surrounding soils (12, 13). For example, when plants absorb NO_3_^−^, they raise the pH, whereas utilizing NH_4_^+^ lowers the pH (12). Thus, plants influence the local pH of the rhizosphere and may magnify or dampen bulk soil pH changes for plant-associated microorganisms (14). In this study, we describe the results of a soil manipulation study in a Christmas tree farm planted with Canaan fir (Abies balsamea) (15). Rhizosphere samples were collected approximately 6 years after the initial sulfur amendment. We endeavored to test if the acidification effect was still present, the influence of the amendment on the nutrient status of the roots, and the composition of the rhizosphere archaeal, bacterial, and eukaryotic communities through sequencing of 16S rRNA and 18S rRNA genes. We additionally investigated the interactions of the rhizosphere communities through co-occurrence networks and the functional potential of the communities through metagenomic sequencing. Through these efforts, we show that a history of soil acidification with elemental sulfur induces significant changes in the fir tree root tissue chemistry as well as the composition and functional potential of the rhizosphere microbial populations.

## RESULTS

### Soil chemistry.

In 2020, 6 years after the initial sulfur treatment, the pH of the acidified soils remained approximately 1.4 pH units lower than that of the control soils (Table 1). Soil acidification was also associated with significantly lower calcium, magnesium, and organic matter contents than in the control soils (Table 1). Thus, the effects of the pH treatment were still apparent during the current study and influenced several other soil parameters.

**TABLE 1:** Bulk soil chemistry^*a*^

Treatment	Mean pH (SD)	Mean Ca concn (ppm) (SD)	Mean Mg concn (ppm) (SD)	Mean P concn (ppm) (SD)	Mean K concn (ppm)	Mean OM (%) (SD)	Mean CEC (meq/100 g) (SD)
Control	6.5 (±0.1)	1,997 (±207)	88 (±2)	512 (±54)	94 (±13)	4.5 (±0.3)	10.7 (±0.8)
Acidified	5.1 (±0.1)***	619 (±170)**	49 (±7)**	532 (±44)	88 (±8)	3.8 (±0.1)*	12.1 (±0.6)

a Values represent the means from triplicate measurements, with standard deviations indicated in parentheses. Significance determined by a nonpaired *t* test is denoted by asterisks (*, *P* ≤ 0.05; **, *P* ≤ 0.01; ***, *P* ≤ 0.001). OM, organic matter; CEC, cation exchange capacity.

### Root tissue analysis.

**FIG 1:**
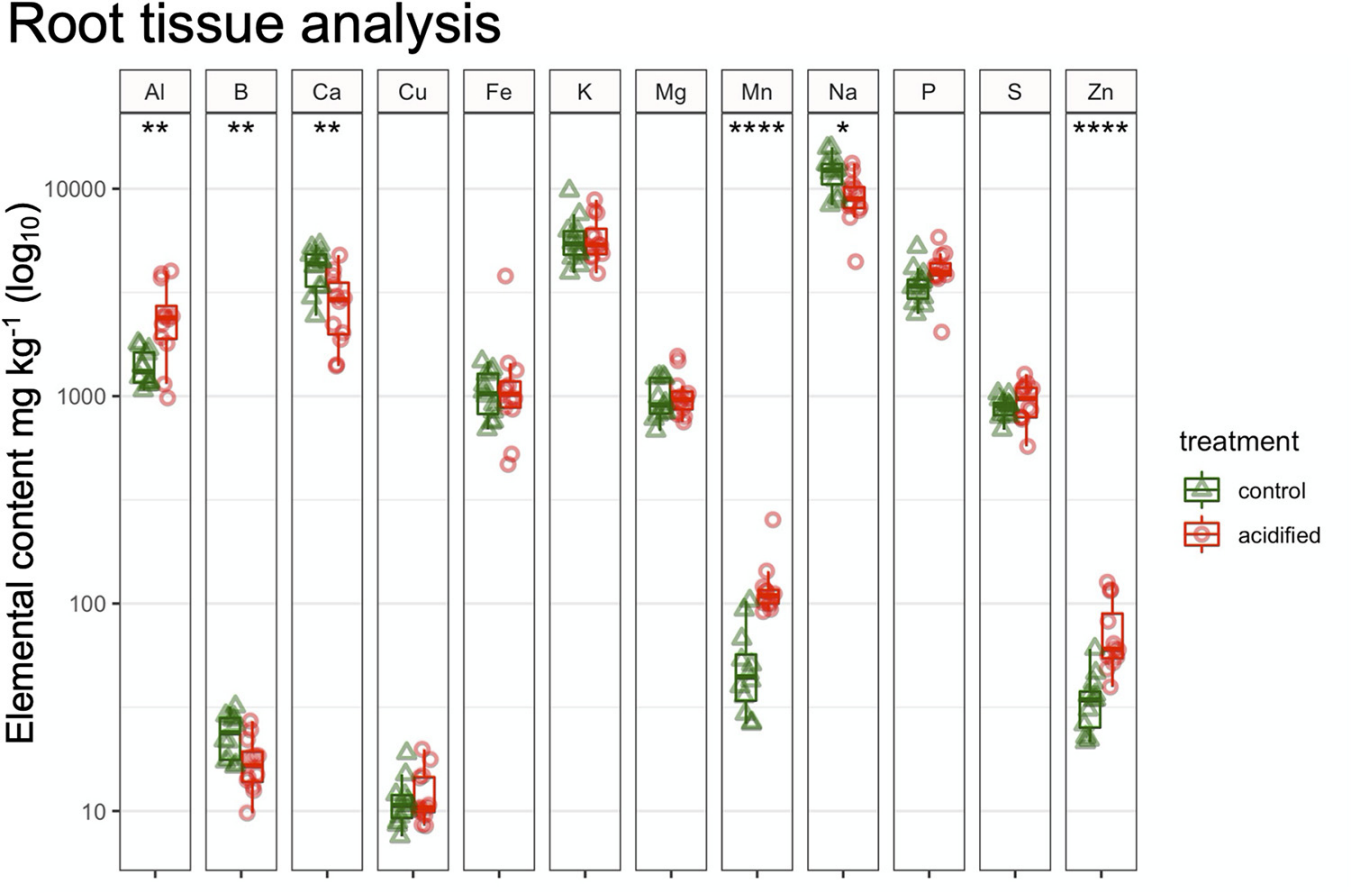
ICP-OES analysis of root tissue. Note that the roots were washed in a solution of phosphate-buffered saline, which explains the high Na, P, and K values (see Materials and Methods for the formula). Thus, these values should be considered only relative to the acidification treatment. Significance determined by a *t* test is denoted by asterisks (*, *P* ≤ 0.05; **, *P* ≤ 0.01; ****, *P* ≤ 0.0001).

The mineral nutrition status of the root tissue was analyzed by inductively coupled plasma optical emission spectroscopy (ICP-OES). Three elements, B, Ca, and Na, were present at significantly lower concentrations in roots from the acidified soils (36, 47, and 31% lower, respectively), whereas Al, Mn, and Zn were significantly more abundant in the roots from the acidified soils (56, 42, and 47% higher) (Fig. 1). Note that prior to the ICP analysis, the roots were rinsed with phosphate-buffered saline (PBS) to remove rhizosphere soil. This procedure may have influenced the measured values for Na, P, and K, and so the values that we report for these elements should be considered relative rather than absolute values in the root tissue for comparisons between the roots in control and acidified soils. Yet these data clearly demonstrate that the acidification treatment of the soils translated into altered chemistry of the fir tree root tissue. Interestingly, the sulfur levels were not significantly different between roots from control and acidified soils, suggesting that pH effects may have outlasted any influence of elevated sulfur in the soils.

### Diversity of 16S rRNA and 18S rRNA gene libraries.

**FIG 2:**
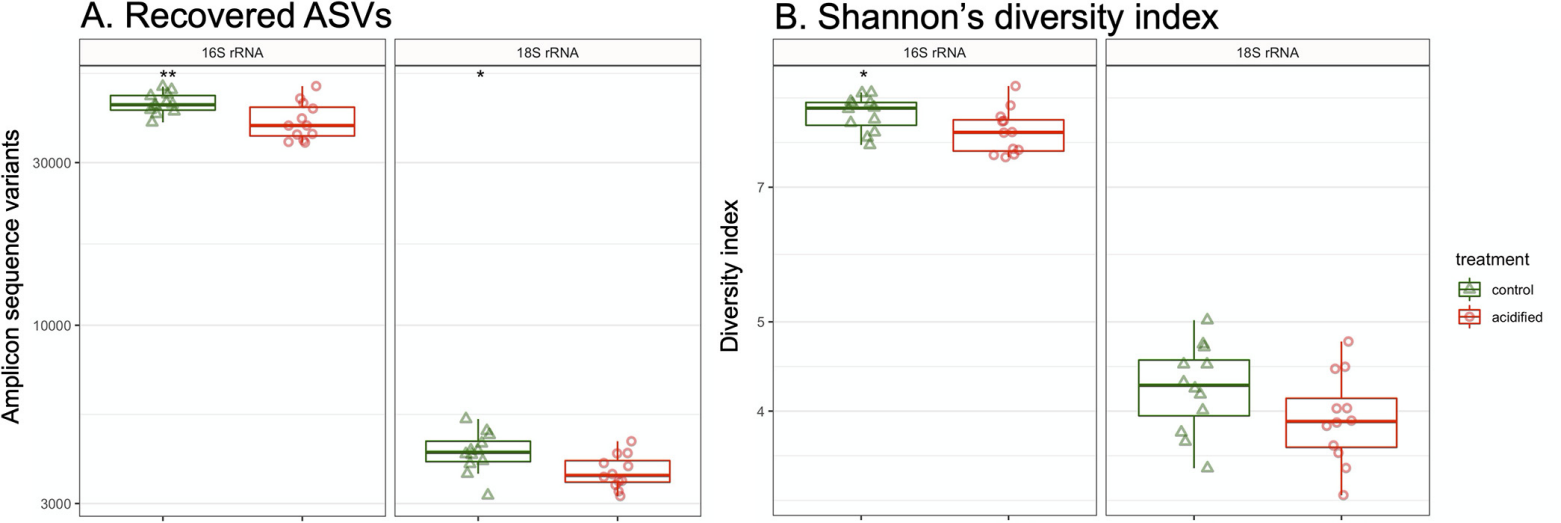
Alpha-diversity of 16S rRNA and 18S rRNA gene data sets. (A) Number of recovered ASVs. (B) Shannon’s diversity index based on ASV relative abundance. For both panels, significance determined by a *t* test is denoted by asterisks (*, *P* ≤ 0.05; **, *P* ≤ 0.01).

Sequencing of the 16S rRNA gene was pursued to investigate the bacterial and archaeal populations in the fir tree rhizosphere, whereas 18S rRNA gene sequencing was used to characterize the eukaryotic populations. The alpha-diversity of the 16S and 18S rRNA gene libraries was assessed to determine if the acidification treatment influenced the community diversity of the fir tree rhizosphere. A statistically higher number of 16S rRNA gene amplicon sequence variants (ASVs) was recovered from roots in the control soils (mean, 44,830 ASVs) than in the acidified soils (39,868 ASVs), an 11% decrease in the number of recovered ASVs (Fig. 2A). Similarly, Shannon’s diversity index of the 16S rRNA gene libraries was significantly higher in the control soils (Fig. 2B). For the 18S rRNA gene libraries, a mean of 4,266 ASVs were recovered from the control rhizospheres, versus a mean of 3,734 ASVs from roots in the acidified soils (Fig. 2A), a 12% decrease in association with the acidification sulfur treatment. Yet for the 18S rRNA gene libraries, Shannon’s diversity indices were similar between the control and acidified samples (Fig. 2B). Taken together, these data suggest that the 16S rRNA gene data sets were more diverse than the 18S rRNA gene data sets, pointing to diverse bacterial/archaeal communities in comparison to their eukaryotic counterparts. Additionally, the treatment was associated with a significant decrease in the number of recovered ASVs for both the 16S and 18S rRNA gene libraries, suggesting that soil acidification resulted in a trend toward decreased diversity of both the 16S rRNA and 18S rRNA gene amplicon data sets.

### Alterations in 16S rRNA libraries due to soil acidification.

**FIG 3:**
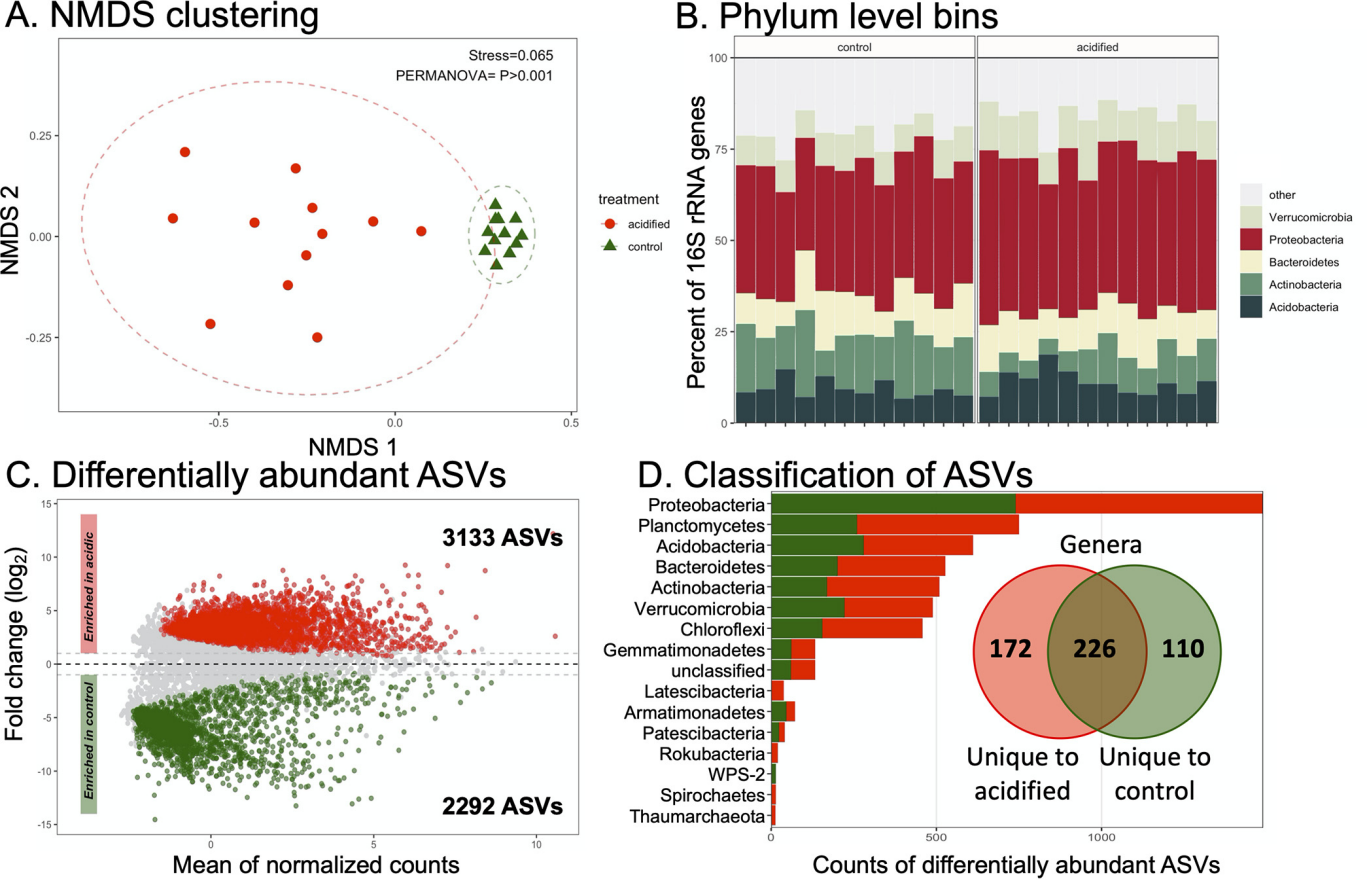
Composition of 16S rRNA gene data sets. (A) Nonmetric multidimensional scaling ordination on ASV-level data. The stress value of the ordination and *P* value from statistical permutational multivariate analysis of variance (PERMANOVA) are indicated. Ellipses denote 95% confidence intervals fitted onto the ordination. (B) Phylum-level taxonomic bins in the data sets. Phyla consistently accounting for >1% of sequence reads are displayed, with the remainder assigned to the category “other.” (C) MA plot displaying differentially abundant (DA) ASVs. The numbers of DA ASVs enriched under each condition are indicated in the inset text. (D) Taxonomic classification of DA ASVs. Each bar represents the sum of DA ASVs classified into each phylum. The inset Venn diagram shows ASVs classified to the genus level. The diagram shows the sum of genus-level bins uniquely enriched in the control or acidified soils, with the overlap indicating genera with members enriched under both conditions.

The relationship between sequence data sets was visualized with nonmetric multidimensional scaling (NMDS) and showed that the control data sets clearly clustered distinctly from the amended samples, with a *P* value of ≤0.001 (Fig. 3A). Communities for each tree with acidified soil had greater intersample distances in the NMDS plot, suggesting that the soil sulfur treatment inflated community heterogeneity among individual rhizosphere samples. This was confirmed by comparing the intersample dissimilarities between samples, which were larger for the data sets from acidified soil and were highly significant (*P* ≤ 0.0001) (see Fig. S1 in the supplemental material).

The 16S rRNA gene sequences were classified to the phylum level. A total of 37 phyla were identified in the data set, with the 5 most abundant phyla generally making up >75% of sequence reads (Fig. 3B). Overall, the relative abundances of phyla were similar between the control and acidified rhizospheres. Yet 20 phylum-level bins were identified as significantly different in relative abundance due to the pH treatment (Table S3). For instance, an increase of *Proteobacteria* was associated with acidification, rising from 35% of the control sequence libraries to 42% in the pH treatments. Taken together, these data show that the pH treatment was associated with shifts in the taxonomic composition of the rhizosphere communities at broad taxonomic levels.

**FIG 4:**
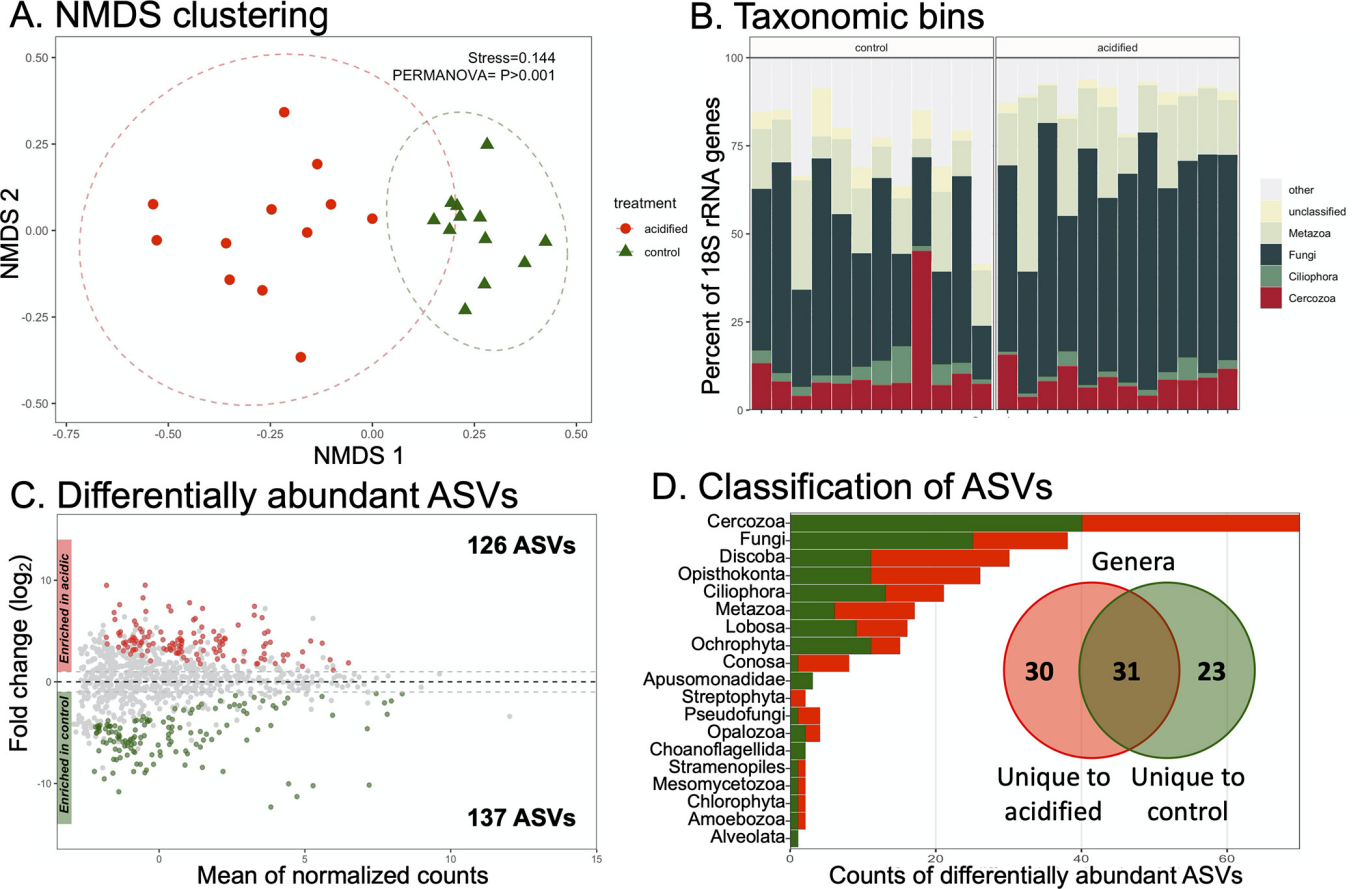
Composition of 18S rRNA gene data sets. (A) Nonmetric multidimensional scaling ordination on ASV-level data. The stress value of the ordination and *P* value from statistical PERMANOVA are indicated. Ellipses denote 95% confidence intervals fitted onto the ordination. (B) Taxonomic bins in the data sets. Taxa consistently accounting for >1% of sequence reads are displayed, with the remainder assigned to the category “other.” (C) MA plot displaying differentially abundant (DA) ASVs. The numbers of DA ASVs enriched under each condition are indicated in the inset text. (D) Taxonomic classification of DA ASVs. Each bar represents the sum of DA ASVs classified into each taxon. The inset Venn diagram shows ASVs classified to the genus level. The diagram shows the sum of genus-level bins uniquely enriched in the control or acidified soils, with the overlap indicating genera with members enriched under both conditions.

We additionally tested for significant differences in ASV relative abundances due to soil acidification. Thousands of ASVs were found to be significantly different in response to the pH treatment (Fig. 3C). The differentially abundant (DA) ASVs belonged to 15 different phyla (Fig. 3C). Most of the DA ASVs belonged to the phylum *Proteobacteria*, matching their dominance in the data sets (Fig. 3B). However, the second-largest class of DA ASVs was the *Planctomycetes*, which were among the rare “other” phyla in the rhizosphere (Fig. 3B and D). Yet *Proteobacteria*- and *Planctomycetes*-related ASVs were identified as being enriched in both the control and acidified soils, suggesting that there was not a consistent response across the groups. There were some DA ASVs that belonged to phyla specifically enriched in the acidified soils, namely, the *Latescibacteria*, *Rokubacteria*, and *Spirochaetes* phyla and the archaeal phylum *Thaumarchaeota*. Similarly, WPS-2-related DA ASVs were unique to the control soils (Fig. 4D). These observations are likely influenced by the relative rareness of these taxa in the data sets (Fig. 3B) and thus may not be a true reflection that these taxa are particularly sensitive to the acidification treatment. The DA ASVs could be further classified into 508 different genus-level bins (Fig. 4D, inset Venn diagram). An interesting observation was that a large proportion of the differentially abundant genus-level bins (226; 44%) contained genera with ASVs that were identified as being more abundant in both the control and acidified soils. This suggests that much of the response to acidification was occurring at a subgenus level, i.e., between species of the same genus or even specific ASVs. Taken together, these data show that a diverse set of taxa was identified as responding to the soil acidification treatment and that few taxa showed particular sensitivity to soil acidification in one direction or the other.

### Alterations in 18S rRNA libraries due to soil acidification.

Assigning 18S rRNA genes to ASVs and visualizing sample relatedness by NMDS demonstrated a significant independent clustering of the control and acidified data sets (*P* < 0.001) (Fig. 4A). However, contrary to the 16S rRNA data sets, the average pairwise distances between control and acidified samples were not significantly different (Fig. S1). This suggests that heterogeneities between populations were similar between the control and acidified rhizospheres for the eukaryotes.

The 18S rRNA genes were classified to explore the relative abundances of taxa in the data sets (Fig. 4B). There were wide variations in the average relative abundances of 18S rRNA taxonomic bins between the control and acidified soil data sets. For example, the percentage of sequences related to the Fungi increased from an average of 39% in control samples to 56% in the acidified soils, yet the differences were not significant. In fact, only a single taxonomic bin, the Conosa (a subphylum of the Amoebozoa), was identified as significantly different between the control and acidified soils, being more abundant in the control soils (Table S3).

A multitude of 18S rRNA ASVs were identified as DA due to the acidification treatment (Fig. 4C). The DA ASVs belonged to 15 different taxonomic ranks, with 4 unclassified bins (Fig. 4D). The DA ASVs could be further classified into 84 genus-level bins (Fig. 4D, inset Venn diagram). One observation of note was that the largest proportion of DA ASVs belonged to the group Cercozoa (Fig. 4D), although they were not particularly abundant across the data sets (Fig. 4B). This suggests that these organisms may be particularly sensitive to the acidification treatment among the eukaryotes. Yet the Cercozoa often act as bacterivorous predators, with different feeding strategies (16). In this regard, it is unclear if the Cercozoa are responding to the soil acidification *per se* or an alteration of their prey bacterial populations with acidification. In any case, the data shown here also support that a wide array of the eukaryotic rhizosphere communities were sensitive to changes in soil pH; whether this was a response to environmental conditions or changes in the plant health status or bacterial communities remains to be demonstrated.

### Network analysis.

**FIG 5:**
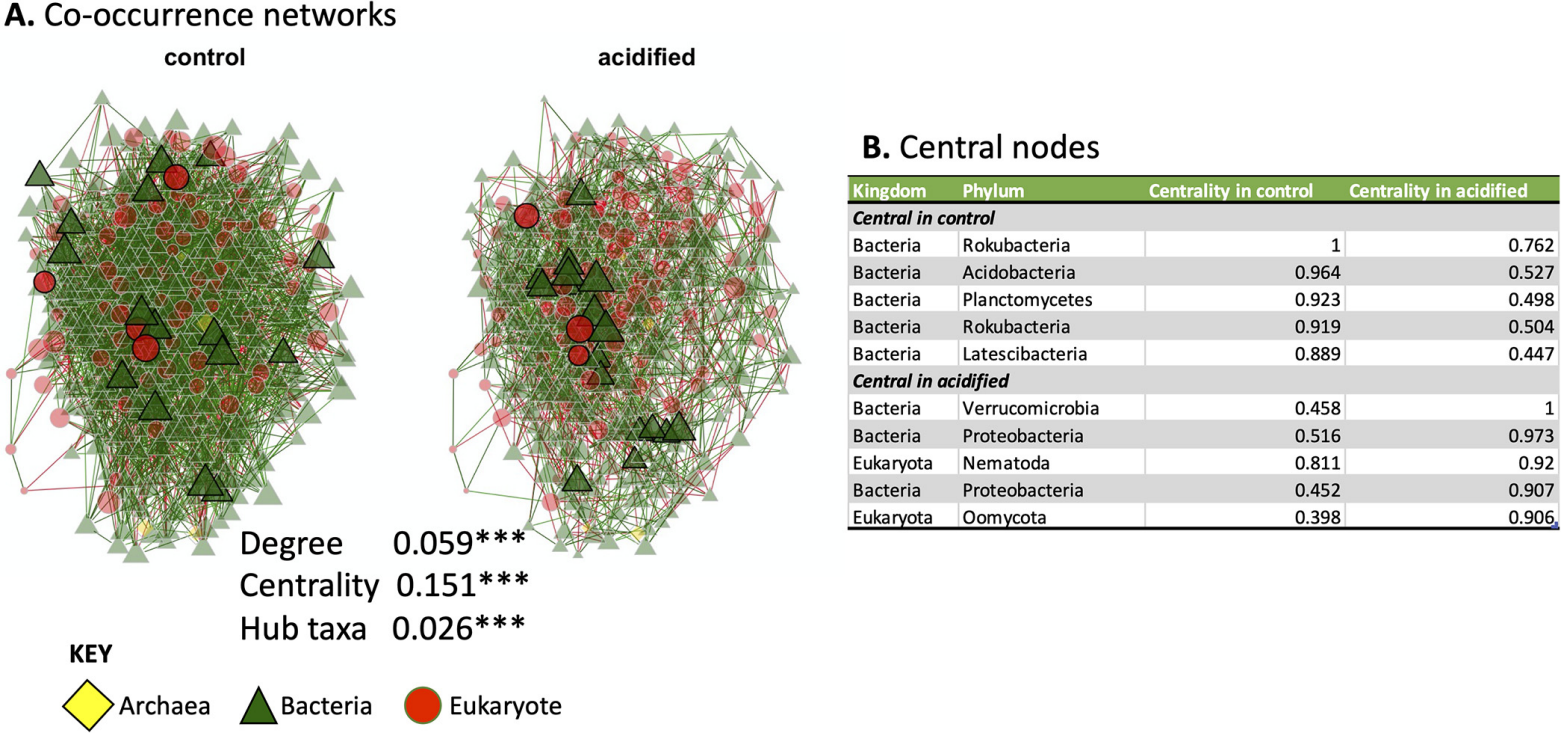
(A) Network analysis. Each node represents an ASV colored and shaped by the kingdom to which it belongs. Network connections are colored by their association direction. Positive associations are in green, and negative associations are in red. The size of the nodes denotes the centrality of the taxon, with larger nodes having the most connections. The absolute differences in degree, eigenvector centrality, and hub taxon measures between the two networks are indicated. Each value was statistically significantly different with a *P* value of ≤0.001 (indicated by the asterisks). (B) List of the top five most central nodes in the control and acidified soils. Each line represents an individual ASV. The kingdom- and phylum-level classifications of each ASV are indicated. The eigenvector centrality, which was used to rank the centrality of the ASVs, is also displayed.

Co-occurrence networks were constructed to characterize bacterial, archaeal, and eukaryotic interactions. To focus on the most abundant ASVs, only those ASVs with sequence counts of ≥50 and that were present in both control and acidified data sets were retained. The resulting data set consisted of 317 16S rRNA gene ASVs and 79 18S rRNA gene ASVs. The co-occurrence network is diagrammed in Fig. 5A. Qualitative differences in the network structure are readily apparent from inspecting the network structure. Quantitatively, the adjusted Rand index between the two networks was 0.016 (*P* < 0.001 by a two-tailed *t* test), suggesting that there were highly significant differences in the network topologies between the control and acidified soils. Similarly, measurements of network degree (number of connections), eigenvalue centrality (connectedness of nodes), and the hub taxa (taxa that are most connected to other taxa, similar to “keystone” species) all significantly differed between the two networks (Fig. 5). Finally, the most central hub taxa were identified for each network (Fig. 5B). All of the 5 most central taxa in the control network were bacteria, whereas two eukaryotes are present in the central taxa of the acidified soil network. In this regard, these data show that the soil acidification treatment altered not only the structure of the soil community but also how taxa interact and the keystone species that support the community.

### Metagenomic sequencing and functional potential of the rhizosphere communities.

**FIG 6:**
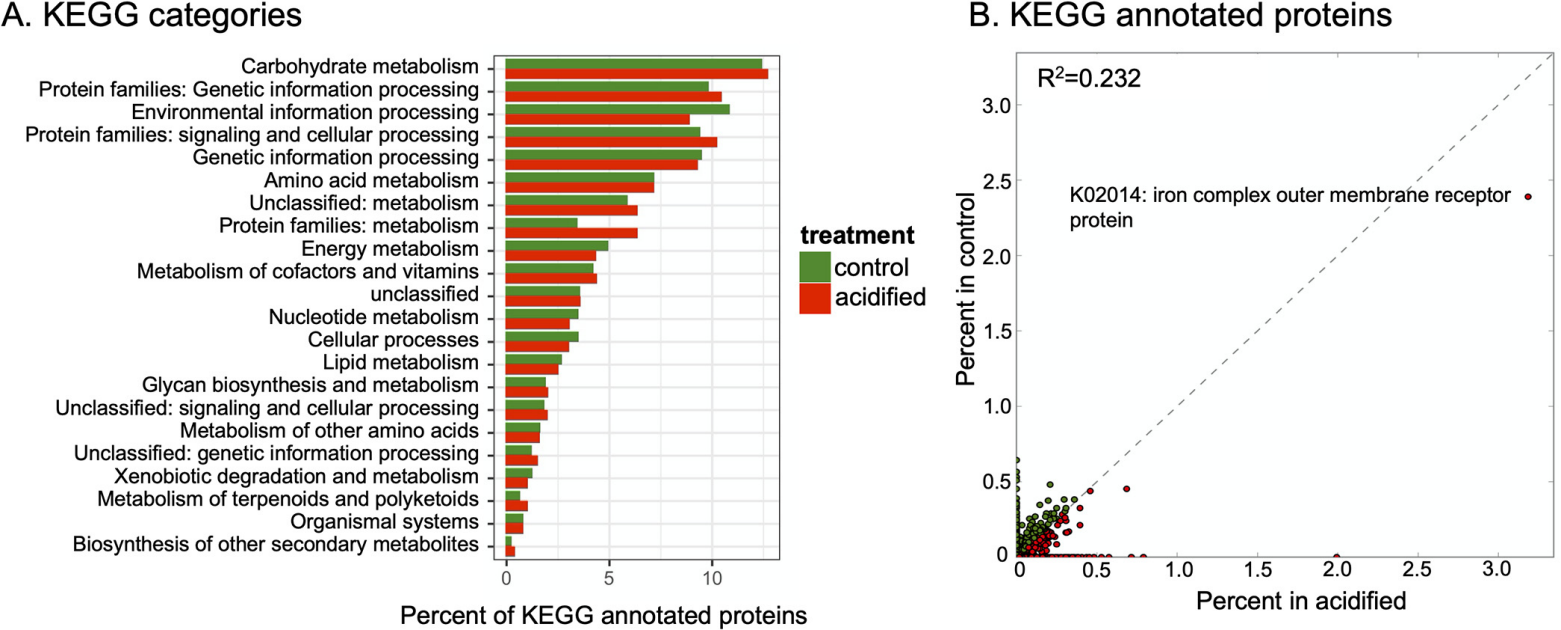
Functional profile of genes in the metagenomes. (A) Relative abundance of functional categories in the KEGG database. (B) Relative abundance of KEGG protein annotations. The identity of the most abundant protein in the data sets is indicated.

**FIG 7:**
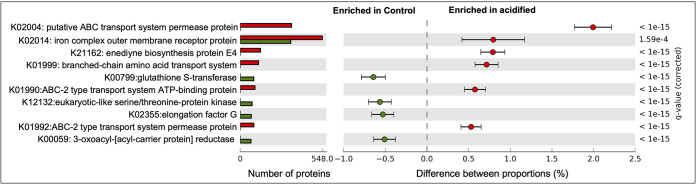
Differentially abundant KEGG-annotated proteins in the metagenomes. The numbers of proteins identified for each KEGG annotation are shown in the bar graph. The differences in the proportions of each protein and estimated 95% confidence intervals are also displayed.

Bulk sequencing of metagenomic DNA was undertaken to describe the functional gene repertoire of the fir tree rhizosphere communities. Totals of 127,547 and 157,542 genes were identified in the control and acidified metagenomes, respectively. Of these genes, 25.8% and 24.4% were functionally annotated, for a total of 32,903 and 38,469 identified proteins in the control and acidified metagenomes. The proteins were assigned to KEGG functional categories, and the metagenomes displayed similar compositions (Fig. 6A). For instance, categories related to carbohydrate metabolism, genetic information processing, and environmental information processing were predominant in both metagenomes and generally present in similar proportions (Fig. 6A). At the individual-protein level, most identified proteins were in relatively low abundance (<0.5% of identified proteins) and present in the two metagenomes in similar relative abundances (Fig. 6B). The most abundant protein in both data sets was identified as an iron complex outer membrane receptor protein, which participates in iron uptake from the environment (17). Differentially abundant proteins in the two metagenomic data sets were identified, and 10 proteins were documented with at least a 0.5% difference in relative abundance and a *P* value of <0.05 (Fig. 7). Of the identified proteins, five were involved in transport processes: three ABC transporters, the iron transporter identified as the most abundant protein in the data sets (Fig. 6B), and a branched-chain amino acid transporter (Fig. 7). Of note is that all five transporter proteins were more abundant in the acidified metagenome, potentially indicating more genes for scavenging resources in low-pH soils. As these data are from unreplicated semiquantitative metagenomic sequencing, the results should be interpreted with caution. However, these limited data point to functional changes in the soil microbial communities, particularly related to transport processes.

## DISCUSSION

Soil pH has been identified as a master variable controlling nutrient availability and plant productivity in agricultural soils (18). Here, we observe that the soil acidification treatment with elemental sulfur led to altered mineral nutrient status for the fir tree roots, which was still present 6 years after the initial treatment. Specifically, the concentrations of Al, Mn, and Zn in the root tissue were significantly higher in root tissues from the acidified soils than in the controls (Fig. 1). Al is generally abundant in soil but is not considered to be an essential nutrient for plants, and even micromolar amounts of Al in root tissue can be toxic (19, 20). Al toxicity can alter root morphology, lead to deficiencies in other nutrients such as Ca, and cause genetic damage through interactions with plant cellular DNA (reviewed in reference 21). In comparison, Mn and Zn are required components of the photosynthesis proteins in plants and are thus considered essential nutrients (22). Yet both nutrients also show toxic effects when provided in excess (23). Thus, these data point to potential stressors in the root tissue related to metal toxicity. In contrast, the levels of B and Ca were reduced in root tissues (Fig. 1). Both B and Ca play roles in plant cell wall synthesis, and deficiencies are associated with poor plant health (24–26). It is important to note that the trees in the field, including those in the acidified soils, did not show any observable symptoms of phytotoxicity or nutrient deficiency. Indeed, the Canaan fir in this trial is a species adapted to growing in very acidic soil; the trees in the acidified plots have shown better growth and color than those growing at a higher soil pH (27). Yet these observed differences in root mineral nutrient status in association with soil acidification are likely to alter root physiology, which presumably could translate into the alterations of the root-associated microbial community in the rhizosphere.

Multiple lines of evidence support that the rhizosphere communities were significantly and dramatically altered in association with soil acidification. Lower soil pH was associated with reductions in alpha-diversity of both the 16S rRNA and 18S rRNA gene data sets, indicating that both the bacterial/archaeal and eukaryotic populations were less diverse in the lower-pH soils investigated here, although the effect and significance were greater for the bacterial/archaeal 16S rRNA gene data sets than for the 18S rRNA genes (Fig. 2). Multiple studies have reported a reduction of alpha-diversity for microbial populations under soil acidification (28). Thus, a reduction in diversity under soil acidification appears to be a common phenomenon across multiple soil environments.

NMDS clustering, differential abundances of taxonomic bins and ASVs (Fig. 3 and 4), and network analysis (Fig. 5) all pointed to an altered microbial community structure in the rhizospheres from control soils in comparison to their acidified counterparts. While other studies have identified particular taxa such as the *Acidobacteria* and *Actinobacteria* as being particularly responsive to soil acidification (14, 29), the data presented here suggest a more generalized response across the community. There was not a strong taxonomic signal in the differentially abundant organisms identified. In the 16S rRNA data sets, multiple phylum-level taxonomic bins and thousands of ASVs belonging to hundreds of individual genera shifted in abundance due to decreased pH (Fig. 3). Similar patterns were observed in the 18S rRNA data sets (Fig. 4). This broad taxonomic response is likely related to the observation of increased heterogeneity in the rhizosphere communities in the acidified soils, particularly for the 16S rRNA gene data sets (Fig. 3A; see also Fig. S1 in the supplemental material). It has long been recognized that the “coefficient of variation” is a useful tool to assess the stability of ecological communities, where increased variability is a signal of reduced ecological stability (30). This potential ecosystem instability is also reflected in the co-occurrence networks where the taxa in the acidified soils are less connected and display lower centrality values (Fig. 5). In this respect, we propose that the acidified soils may not yet have converged on a stable state, even 6 years after disturbance. Alternatively, the addition of pelletized sulfur to the soil may have simply increased the environmental heterogeneity of the soil, resulting in pH hot spots and cold spots, translating into an elevated intersample divergence. Of course, these hypotheses are not mutually exclusive. Yet taken together, these data suggest that a large effect of the soil acidification was a generalized increase in the heterogeneity among the rhizosphere communities, including archaea, bacteria, and eukaryotes, rather than a targeted enrichment or depletion of specific populations.

Metagenome sequencing provided insight into how the taxonomic alterations of the soil communities translated into functional potential. Only a minority of the proteins identified in the data sets were able to be annotated (∼25%), which is similar for other soil metagenomes (31). At the broadest classification, the metagenomes contained a similar functional profile predominantly harboring genes for carbohydrate metabolism, genetic information processing, and environmental sensing (Fig. 6A). Yet at deeper annotation levels, the acidified metagenomes appeared to harbor more genes for the transport of various compounds. Bacteria in the soil are dependent on minerals in the environment to meet their nutritional needs. As shown in Table 1 and Fig. 1, the soil acidification treatment was associated with large state changes in mineral and organic contents in both the bulk soil and plant roots. Thus, the elevated number of transport-related genes in the metagenomes from the acidified soils may indicate an increased ability to scavenge resources from their surroundings (32). In contrast, levels of toxic elements such as Al and Zn were elevated in plant root tissue (Fig. 1); therefore, transporters may also play a role in detoxifying the local environment (33). Additionally, pH homeostasis in microorganisms may require active pumping of protons out of the cell (34). Thus, elevated levels of transport proteins in the metagenomes from the acidified soils may represent a physiological adaptation to low pH. Due to the low resolution and replication of these metagenomic data sets, these observations will need to be followed up with more quantitative techniques. Yet they point to some of the potential adaptations that allow microbes to inhabit soils that differ in pH.

### Conclusion.

The data presented here demonstrate that the effects of soil acidification are manifest across the range of organisms that inhabit the soil. These include changes in the mineral nutrient status of the host plant as well as compositional alterations in the associated archaeal, bacterial, and eukaryotic communities that populate the rhizosphere soils. The microbial communities were less diverse in the acidified soils, and alterations in the taxonomic composition of the communities were evident at multiple taxonomic ranks, suggesting that a wide variety of members of the soil microbial community were affected by the acidification treatment. These taxonomic shifts resulted in an increased heterogeneity in community structure among the microbial populations in the acidified soils. This could indicate that the acidified soils are in a transitional state or inhabit an environment with increased spatial heterogeneity. Finally, metagenome sequencing demonstrated that the taxonomic reshaping of the community translated into alterations in the functional potential of the indigenous rhizosphere populations. These data underscore the importance of soil pH as a driving force in determining the structure and function of soil communities and highlight the critical research need to integrate plant and microbial responses in the rhizosphere and their responses to soil acidification.

## MATERIALS AND METHODS

### Field site description.

Christmas trees of the species *Abies balsamea* (L.) Mill. var. *phanerolepis* Fernald (Canaan fir) were located in the Allen Hill Farm in Brooklyn, CT (41.7696, −71.9183). Plots were 3.4 by 11.7 m, planted with 14 trees at a spacing of 1.7 m between trees, with a similar spacing of 1.7 m between rows. On 18 June 2014, pelletized sulfur (containing 90% sulfur) was applied to the plots at a rate of 3,370 kg per ha. The sulfur was incorporated into the soil mechanically with a rototiller to a depth of 15 cm. Three-year-old 30-cm-tall root transplants were planted in the field on 13 and 14 April 2015. Soil pHs were measured in August 2015 and were 4.1 and 5.9 for the acidified and control soils, respectively. Samples for this study were collected on 2 June 2020, almost 6 years after the soil sulfur amendment was initially performed.

### Soil chemistry.

Soil cores were collected to a depth of 10 cm from between trees within the same row. Three cores were collected per row and were composited into a single sample. Three replicate rows were sampled for both the control and amended soils, resulting in three independent replicates for soil chemical analysis. Soil chemistry was performed with the Ag Soil test (Spectrum Analytic) using standard methods.

### Root collection.

Fine roots were uncovered with an ethanol-sterilized trowel in the vicinity of an individual tree. Rhizosphere samples were processed in a manner similar to that described previously by McPherson et al. (35). Briefly, six ∼10-cm sections of fir tree fine roots were collected from each tree and shaken vigorously to remove loosely adhering soil. The root sample was then transferred to a 50-ml plastic centrifuge tube containing 25 ml of sterile phosphate-buffered saline (PBS) (ingredients of PBS [by weight] in 1 liter of deionized water were 8 g of NaCl, 0.2 g of KCl, 1.44 g of Na_2_HPO_4_, and 0.24 g of KH_2_PO_4_). The samples were then stored on ice until further processing in the field. Roots were collected from 4 individual trees per row, with 3 rows sampled per soil treatment, resulting in 12 rhizosphere/root samples per soil treatment. The rhizosphere soil was removed from the roots in the field by vortexing the root samples for 2 min at full speed. The roots were then removed with ethanol-sterilized forceps and transferred to a sterile plastic sample bag. The soil remaining in the centrifuge tube after vortexing was considered the rhizosphere sample and was immediately stored on dry ice. Root and rhizosphere samples were transported to the laboratory in New Haven, CT. The rhizosphere samples were stored at −80°C, while the root samples were stored at −20°C until further processing.

### Root nutrient analysis.

To analyze elemental content in the roots, tissues were oven dried at 65°C for 48 h. About 0.2 g per dried sample was digested with 4 ml of concentrated nitric acid (∼70%) for 45 min at room temperature and for an additional 45 min at 115°C in a hot block (DigiPREP system; SCP Science, Champlain, NY). After cooling, the final volume was fixed with deionized water. The elemental profile of the digests was determined by inductively coupled plasma optical emission spectroscopy (ICP-OES) (iCAP 6500; Thermo Fisher Scientific, Waltham, MA). Yttrium was employed as an internal standard, and a multielement sample of known concentrations was included in the run for quality control purposes.

### Rhizosphere DNA extraction.

The frozen rhizosphere samples were removed from the −80°C freezer and rapidly thawed in a 55°C water bath. Soil particles and cells were collected by centrifugation at 14,000 rpm for 10 min at 4°C. DNA was extracted from 0.25 g of soil from the resulting pellet using the DNeasy PowerSoil kit (Qiagen). DNA extractions were verified by gel electrophoresis in a 1% agar gel.

### Amplification of 16S rRNA and 18S rRNA genes.

Bacterial and archaeal 16S rRNA genes were amplified with the primer pair 515F (GTGYCAGCMGCCGCGGTAA) and 806R (GGACTACNVGGGTWTCTAAT) (36). Extracts were each amplified with 10 μl Platinum SuperFi II DNA polymerase (Invitrogen), which also included 7.5 μM both the mPNA and pPNA peptide nucleic acid (PNA) clamps (mPNA, GGCAAGTGTTCTTCGGA; pPNA, GGCTCAACCCTGGACAG) to block the amplification of host plant mitochondria and plastid rRNA genes, respectively (37). PCR conditions consisted of 94°C for 2 min followed by 30 cycles of 94°C for 15 s, 60°C for 15 s, 68°C for 15 s, and 4°C for an infinite hold. The resulting amplification products were verified by gel electrophoresis, and cleaning and normalization of individual PCR products were performed with a SequalPrep normalization plate (96) kit (Invitrogen). The normalized PCR amplicons were mixed, and the quantity and quality of the DNA pool were verified using an Agilent TapeStation. The resulting 16S rRNA gene amplicons were submitted to the Yale Center for Genome Analysis for sequencing on the Illumina MiSeq platform using 2- by 250-bp chemistry.

For eukaryotic 18S rRNA gene amplification, we first undertook to design a method to block the amplification of 18S rRNA genes from the host tree. A new PNA clamp was designed based on the pipeline described previously by Taerum et al. (27). To obtain a reference 18S rRNA sequence for PNA clamp design, DNA was extracted from A. balsamea needles using a GeneJET plant genomic DNA purification minikit (Thermo Scientific) according to the manufacturer’s directions for DNA purification from lignified polyphenol-rich plant tissues. A 170-bp fragment of the 18S rRNA gene was amplified with the primers Euk1391F (5′-GTACACACCGCCCGTC-3′) and EukBr (5′-TGATCCTTCTGCAGGTTCACCTAC-3′) (38). This fragment includes the V9 hypervariable region, which is one of the most frequently targeted regions for high-throughput sequencing of eukaryotes. PCR was performed as described previously by Taerum et al. (27). The fragment was sequenced at the Keck DNA Sequencing Facility at Yale on an Applied Biosystems 3730xL DNA analyzer.

PNA clamp design consisted of *in silico* fragmentation of the *A. balsamea* V9 sequence into 15- to 17-bp k-mers, which were then mapped to the SILVA database containing animal, plant, and protist sequences (39). k-mers that did not match any animal, plant, or protist sequences were then screened using PNA TOOL (https://www.pnabio.com/support/PNA_Tool.htm) to ensure that the clamp had a melting temperature of between 76°C and 82°C and consisted of fewer than 35% guanines and 50% purines. The selected clamp (AbiesV9_01, with the sequence GTTCGCCGTCTTCGACG) was synthesized by PNA Bio, Inc. (Newbury Park, CA, USA).

Quantitative PCR was used to test the effectiveness of the clamp at different concentrations. Reaction mixtures consisted of 1× SsoAdvanced universal SYBR green supermix (Bio-Rad, Hercules, CA, USA), 0.5 μM each primer, and 2 ng of the template for a total reaction volume of 10 ml. AbiesV9_01 was added to the reaction mixtures at a range of concentrations (0, 0.75, 1.5, 3.75, and 7.5 μM), with each reaction mixture concentration being conducted in triplicate. Reactions were conducted on a CFX96 Touch real-time PCR machine (Bio-Rad) thermal cycler and consisted of an initial denaturation step of 95°C for 2 min, followed by 40 cycles of 95°C for 10 s and 60°C for 15 s. A concentration of 7.5 μM was selected as it suppressed the amplification of host 18S rRNA genes and matched the concentration used for 16S rRNA gene amplification.

Rhizosphere 18S rRNA genes were amplified with the primer pair 1391F and EukBr with Platinum SuperFi II DNA polymerase (Invitrogen) along with 7.5 μM the AbiesV9_01 PNA clamp. The reaction conditions were 95°C for 2 min, followed by 30 cycles of 95°C for 15 s, 78°C for 10 s, 60°C for 30 s, and 72°C for 30 s and 4°C for an infinite hold. The 18S rRNA amplicons were cleaned and normalized identically as for the 16S rRNA gene amplicons and submitted to the Yale Center for Genome Analysis for sequencing on the Illumina MiSeq platform using 2- by 250-bp chemistry.

### Amplicon sequence analysis.

Both 16S rRNA and 18S rRNA gene sequences were initially processed using the mothur software package (v. 1.44.2) (40). Quality filtering consisted of generating contigs and selecting for sequences of at least 253 bp in length for 16S rRNA genes and 80 bp in length for the 18S rRNA gene data sets. Chimeric sequences were identified with the VSEARCH algorithm (41) as implemented in mothur, using the most abundant sequences as a reference for chimera detection. All putative chimeric sequences were removed from the data sets. A total of >8,000,000 high-quality 16S rRNA sequences were retained, with an average of 337,456 per sample (Table S1). For the 18S rRNA data sets, >4,000,000 sequences passed quality filtering, with an average of 170,983 per sample (Table S1). The 16S rRNA sequences were classified against the SILVA v132 reference database using the RDP naive Bayesian classifier (42) as implemented in mothur, and sequences identified as belonging to chloroplasts were removed (39). 18S rRNA gene sequences were classified with the PR^2^ database, also using the RDP naive Bayesian classifier, and sequences identified as unclassified eukaryotes were removed (43). The resulting sets of sequences in both data sets were assigned to ASVs, employing a 100% sequence similarity threshold.

The mothur output files were imported into the phyloseq R package for descriptive and statistical analyses (44). Prior to alpha-diversity calculations and NMDS ordinations, the sequence data sets were subsampled (random without replacement) to the size of the smallest data set to maintain equal sampling between data sets. To identify statistically significant differences in phylum taxonomic bins and ASV relative abundances, unnormalized ASV count data were employed. Rare ASVs, consisting of 5 or fewer sequences, present in <20% of the samples were removed. Data were normalized using centered log ratio transformations, and statistically significant differences were identified with the ALDEX2 package (45).

### Network analysis.

Co-occurrence networks were analyzed on ASVs consisting of at least 50 sequences for both the 16S rRNA and 18S rRNA gene sequence data sets. This resulted in a combined data set of 396 ASVs (317 16S rRNA and 79 18S rRNA). Networks were analyzed with the NetCoMi package in the R software suite (46), employing the SPIEC-EASI metric for network construction (47). Associations were estimated with the SPRING approach (48) with default normalization and zero handling settings. The nlambda and replication numbers were set to 100 and 20, respectively.

### Shotgun metagenomic sequencing.

DNA samples from individual trees in the same row (i.e., 4 trees) were composited at an equimolar ratio to produce templates for metagenome sequencing. In this manner, three replicate samples were sequenced for each soil treatment. Sequencing libraries were prepared using a ligation sequencing kit (catalog number SQK-LSK109; Oxford Nanopore) and individually barcoded with the native barcoding expansion (catalog number EXP-NBD104; Oxford Nanopore). Libraries were sequenced for 72 h on the Oxford Nanopore MinION instrument with the MinION flow cell (R9.4.1). Base calling was performed with Guppy software (4.2.2) using the accurate base-calling model. The resulting Fastq files were assembled with Flye assembly software (2.8-b1674), using the metagenome settings (49, 50). A round of assembly polishing was performed with Racon v. 1.4.19 (51) followed by a second round of polishing with Medaka (v. 1.2.3; https://nanoporetech.github.io/medaka/) using default parameters. The resulting contigs were binned with metaBAT2 (52), and the resulting assembly bins were assessed with CheckM for completeness and contamination (53). Assembly and binning of the reads produced 17 bins from the control metagenomes and 25 bins from the acidified soils (Table S2). The average length of the metagenomic bins was 5.4 Mbp, with average completeness and contamination of 27% and 7%, respectively. None of the assemblies met the suggested qualifications of even a medium-quality metagenome-assembled genome (>90% completion and <5% contamination [54]). Thus, for the remainder of the metagenome analyses, the focus was on the genes in the metagenomes. Genes were identified and translated with the CheckM implementation of Prodigal (55), and the resulting amino acid sequences were assigned to KEGG pathways with the GHOSTX Web server (21, 56, 57). Statistically significant differences in the relative abundances of KEGG protein annotations were identified using two-sample Fisher’s exact test with Storey’s false discovery rate (FDR) method as implemented in the STAMP software package (58).

### Data availability.

All 16S rRNA and 18S rRNA amplicon gene libraries and the shotgun metagenome sequences are available in the NCBI SRA under BioProject accession number PRJNA708254. The *Abies balsamea* var. *phanerolepis* 18S rRNA gene sequence is available in GenBank under accession number MW699166.1.
