# Supplementary material for: What’s under the Christmas Tree? A Soil Sulfur Amendment Lowers Soil pH and Alters Fir Tree Rhizosphere Bacterial and Eukaryotic Communities, Their Interactions, and Functional Traits
Source: Microbiol Spectr. 2021 Jul 7;9(1):10.1128/spectrum.00166-21. doi: 10.1128/spectrum.00166-21 (PMC8552644; doi:10.1128/spectrum.00166-21)
Supplement: Supplemental file 1 — Supplemental material. Download SPECTRUM00166-21_Supp_1_seq11.pdf, PDF file, 0.5 MB [file spectrum00166-21_supp_1_seq11.pdf]

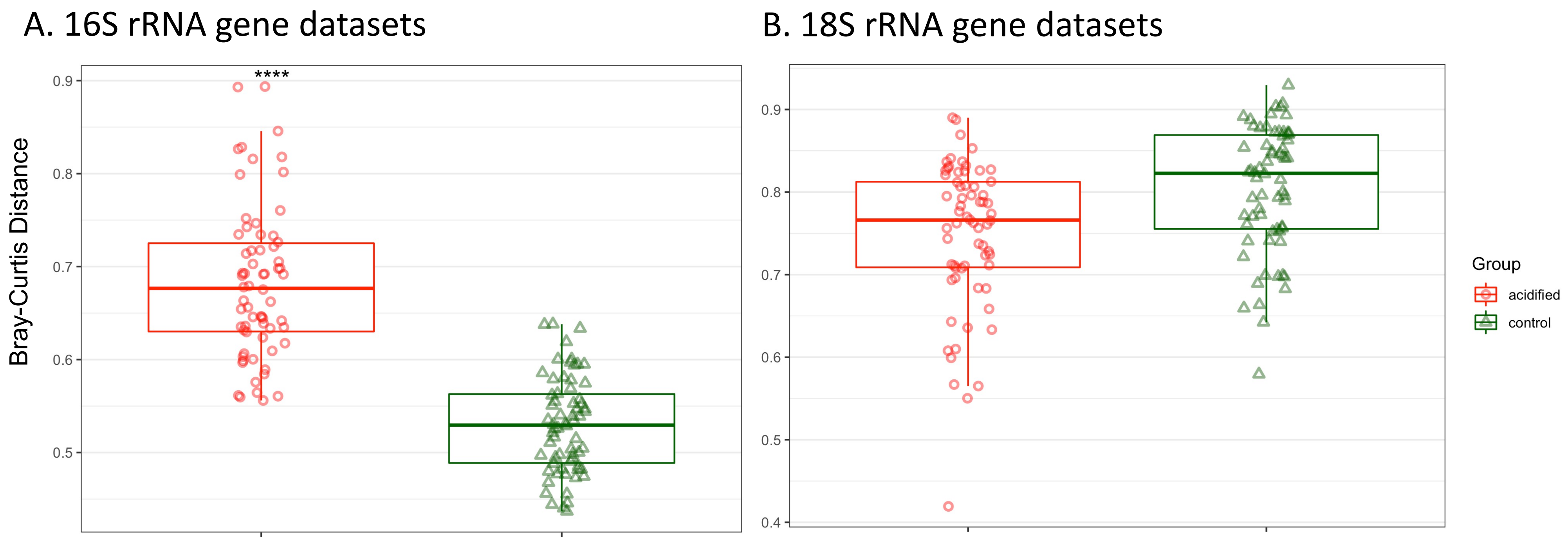

Figure S1. Inter-sample distances. Each box displays the pairwise inter-sample distances between each sample. The acidified soils showed significantly higher inter-sample distances than their control counterparts (P>=0.0001) based on a t-test comparison of means.

Table S1. Sequence recovery for 16S and 18S rRNA gen libraries

16S rRNA gene libraries				18S rRNA gene libraries		
**group**	**Number of sequences**	**ASVs**	**ASVs after subsetting**	**Number of sequences2**	**ASVs2**	**ASVs after subsetting2**
Acidified datasets						
1pHA	290,135	41,482	36,402	145,965	5,314	4,215
1pHB	315,870	55,986	46,253	148,665	4,277	3,400
1pHC	374,167	48,558	34,493	199,711	5,645	3,588
1pHD	**246,600***	50,270	50,270	186,944	6,723	4,564
2pHE	297,192	39,701	34,342	215,996	5,172	3,153
2pHF	368,035	48,461	34,891	146,434	4,398	3,483
2pHG	577,834	75,504	38,536	165,986	4,864	3,497
2pHH	343,362	58,501	44,833	257,999	6,397	3,256
3pHI	317,173	46,914	38,511	190,215	6,007	3,939
3pHJ	294,579	46,417	40,419	178,174	6,061	4,205
3pHK	287,831	41,067	36,256	155,814	4,787	3,658
3pHL	288,625	48,830	43,207	175,226	5,529	3,860
Control datasets						
4CM	724,011	99,408	42,424	**106,851***	4,768	4,768
4CN	312,543	52,813	44,016	178,756	6,262	4,297
4CO	320,978	54,190	44,244	155,373	5,205	3,922
4CP	367,649	53,496	39,370	217,025	6,137	3,670
5CQ	270,826	52,790	49,060	119,885	5,344	4,900
5CR	271,627	50,589	47,060	141,578	6,485	5,309
5CS	251,135	42,230	41,668	161,882	5,665	4,172
5CT	338,639	64,332	50,119	109,391	4,359	4,288
6CU	321,919	54,722	44,429	153,701	4,070	3,169
6CV	248,104	43,084	42,888	246,230	7,544	4,192
6CW	309,398	53,692	45,065	159,846	5,924	4,504
6CX	360,706	63,871	47,614	185,935	6,026	4,000
**Mean**	**337,456**	**53,621**	**42,349**	**170,983**	**5,540**	**4,000**
**Total**	**8,098,938**			**4,103,582**		

*Values in bold represent the smallest dataset to which libraries were subsetted for diversity comparisons Figure 2 in the manuscript.

Table S2. Binning of metagenomic assemblies

Bin	Marker lineage	Accession	Length	Completeness	Contamination
Metagenomic bins from control datasets					
control.13	c__Gammaproteobacteria	(UID4201)	7,065,908	78.03	28.78
control.11	k__Bacteria	(UID203)	8,550,064	67.63	23.1
control.5	k__Bacteria	(UID203)	17,044,403	67.63	87.13
control.17	k__Bacteria	(UID203)	8,218,760	45.23	11.21
control.6	k__Bacteria	(UID203)	30,534,924	44.53	23.99
control.9	f__Flavobacteriaceae	(UID2817)	2,888,290	32.55	0.71
control.8	k__Bacteria	(UID203)	1,410,236	31.58	0
control.14	k__Bacteria	(UID203)	780,725	30.62	0
control.7	k__Bacteria	(UID203)	1,779,157	18.1	0
control.12	k__Bacteria	(UID203)	1,432,572	13.79	0
control.15	k__Bacteria	(UID203)	728,111	9.48	1.72
control.4	k__Bacteria	(UID2570)	292,733	3.89	0
control.3	k__Bacteria	(UID203)	1,249,147	2.59	0
control.2	k__Bacteria	(UID203)	736,014	0.31	0
control.1	root	(UID1)	432,370	0	0
control.10	root	(UID1)	422,726	0	0
control.16	root	(UID1)	16,090,500	0	0
**Average**			**5,862,155.29**	**26.23**	**10.39**
Metagenomic bins from acidified datasets					
pH.13	c__Gammaproteobacteria	(UID4201)	9,893,525	76.86	2.87
pH.4	f__Xanthomonadaceae	(UID4214)	2,754,055	72.17	0.67
pH.3	c__Gammaproteobacteria	(UID4201)	4,549,400	60.51	6.85
pH.19	c__Gammaproteobacteria	(UID4201)	3,762,700	53.75	14.75
pH.18	k__Bacteria	(UID203)	30,624,900	51.57	35.34
pH.14	o__Burkholderiales	(UID4000)	5,773,749	46.26	3.14
pH.6	k__Bacteria	(UID3187)	6,583,557	46.16	8.69
pH.1	k__Bacteria	(UID203)	13,155,322	40.63	17.48
pH.25	k__Bacteria	(UID203)	1,203,107	37.07	0
pH.22	k__Bacteria	(UID203)	9,612,772	35.78	6.61
pH.5	k__Bacteria	(UID203)	12,611,642	33.1	10.34
pH.15	k__Bacteria	(UID203)	1,705,339	26.72	1.72
pH.12	k__Bacteria	(UID3187)	2,776,969	21.4	0
pH.24	o__Burkholderiales	(UID4002)	3,062,998	19.25	0
pH.16	c__Alphaproteobacteria	(UID3305)	1,534,161	17.9	0.87
pH.17	o__Burkholderiales	(UID4000)	1,685,909	11.3	0.08
pH.7	k__Bacteria	(UID203)	816,799	11.08	0
pH.20	k__Bacteria	(UID203)	959,041	8.62	0
pH.21	k__Bacteria	(UID203)	2,410,767	8.25	0
pH.2	k__Bacteria	(UID203)	308,805	7.71	0
pH.10	k__Bacteria	(UID203)	9,023,499	4.08	0
pH.9	k__Bacteria	(UID2982)	859,553	3.2	0.71
pH.8	root	(UID1)	235,137	0	0
pH.23	root	(UID1)	427,163	0	0
pH.11	root	(UID1)	935,356	0	0
**Average**			**5,090,649**	**27.73**	**4.40**
**Overall average**			**5,402,925**	**27.13**	**6.83**

Table S3. Differentially abundant phylum level taxonomic bins due to soil acidification in the 16S rRNA gene datasets.

Phyla	Mean control (% of sequences)	Mean acidified (% of sequences)	Welch's t-test (Benjamini-Hochberg corrected)	Wilcoxon Rank Sum test (Benjamini-Hochberg corrected)
**16S rRNA gene datasets**				
Acidobacteria	9.109	10.894	0.0019	0.0007
Armatimonadetes	0.422	1.138	0.0003	0.0004
Chlamydiae	0.018	0.112	0.0001	0.0002
Cyanobacteria	0.113	0.049	0.0201	0.0191
Dependentiae	0.004	0.017	0.021	0.0037
Elusimicrobia	0.047	0.075	0.0059	0.0112
Entotheonellaeota	0.033	0.005	0.0013	0
Euryarchaeota	0.128	0.026	0.0012	0.0012
FCPU426	0.078	0.269	0.0001	0
Firmicutes	0.043	0.054	0.0128	0.0124
GAL15	0.006	0.001	0.0035	0.0019
Gemmatimonadetes	1.524	1.574	0.0025	0.003
Kiritimatiellaeota	0.005	0	0.0013	0
Latescibacteria	0.633	0.237	0.0022	0.0007
Nitrospirae	0.308	0.171	0.0144	0.0159
Omnitrophicaeota	0.02	0.03	0.017	0.014
Proteobacteria	34.907	41.989	0.0086	0.0066
Rokubacteria	0.968	0.431	0.0076	0.0021
Verrucomicrobia	8.609	11.925	0.0038	0.0024
WPS-2	0.011	0.096	0.0002	0.0001
**18S rRNA gene datasets**				
Conosa	2.060	0.497	0.0005	0.0010
